# Efficacy and safety of tenofovir disoproxil fumarate in preventing vertical transmission of hepatitis B in pregnancies with high viral load

**DOI:** 10.1038/s41598-017-04479-x

**Published:** 2017-06-23

**Authors:** Jun-Ze Chen, Zuo-Wei Liao, Fei-Long Huang, Ru-Kui Su, Wen-Bo Wang, Xue-Yuan Cheng, Jie-Qing Chen, Jia-Qi Liu, Zhong Huang

**Affiliations:** Department of General Surgery, The Ninth Affiliated Hospital of Guangxi Medical University, Beihai, 536000 PR China

## Abstract

This study was a meta-analysis of the literature on the efficacy and safety of tenofovir disoproxil fumarate (TDF) in preventing vertical transmission of hepatitis B in pregnancies with high viral load. Four observational studies and one randomized controlled trial involving 585 pregnant women and 595 newborns were included in the meta-analysis. TDF was more effective than the placebo in reducing vertical transmission in HBeAg-positive chronic hepatitis B (CHB) pregnancies with high serum HBV-DNA levels (OR = 0.21, 95% CI = 0.07–0.61) at 4–12 months, infant HBV DNA seropositivity at delivery (OR = 0.16, 95% CI = 0.07–0.37), and a severe flair in maternal alanine aminotransferase (ALT) levels (OR = 0.43, 95% CI = 0.19–0.95) during pregnancy. In addition, TDF showed more improvement in HBV DNA suppression at delivery (OR = 254.46, 95% CI = 28.39–2280.79). No significant differences were found in HBeAg seroconversion or ALT normalization; or in rates of cesarean section, emergent cesarean section, postpartum hemorrhage, prematurity, congenital malformations, or infant death. However, TDF induced more drug-related adverse events (OR = 2.33, 95% CI = 1.39–3.89) and elevated creatine kinase (CK) (OR = 9.56, 95% CI = 1.17–78.09) than in controls. The available evidence suggests that TDF is effective and safe in preventing vertical transmission of hepatitis B in pregnancies exhibiting a high viral load.

## Introduction

Infection with chronic hepatitis B (CHB) virus remains a major challenge to global health, with approximately 240 million carriers and an estimated > 600,000 deaths annually due to CHB-related disease^1^. Vertical transmission of HBV from hepatitis B surface antigen (HBsAg)-positive and hepatitis B e antigen (HBeAg)-positive pregnancies to infants remains the predominant mode of CHB infection, with up to 90% of infants from these pregnant women becoming chronically infected with HBV without immunoprophylaxis^1–4^, which may be partially due to the active replication and HBeAg positivity of women during their reproductive years^5, 6^. Programs combining postnatal active and passive immunoprophylaxis and universal vaccination have successfully reduced the transmission rates of HBV from > 90% to about 10%^3, 7, 8^; however, there are still about 8%-30% of newborns that fail active HBV immunoprophylaxis (particularly those born to pregnant women with high serum HBV-DNA levels of > 2 × 10^5^–10^7^ IU/mL^8–13^), mainly due to a further increase in the global prevalence of CHB. Thus, preventing vertical transmission of HBV is an effective way to reduce the global burden of CHB.

An increasing number of studies have suggested that antiviral therapies for CHB with perinatal oral nucleotide analogues (NAs) significantly reduce the risk of vertical transmission of HBV in pregnancies with high serum HBV-DNA levels; however, these studies have also shown some conflicting results^2, 3, 8, 10, 12, 14, 15^. In contrast to lamivudine (LAM), telbivudine (LdT) and adefovir (ADV) manifest potency limitations and low threshold to resistance, and TDF is the only approved NA showing greatest potency but without any associated clinical resistance^16, 17^. The favorable efficacy and safety profile of TDF have been well demonstrated for the treatment of HIV mono-infected and HIV/HBV co-infected mothers^10, 18–20^, but the data on the use of TDF in HBV mono-infected pregnancy are limited. Since Pan *et al*. published their first case series in 2012^10^ where they evaluated the efficacy and safety profile of TDF in HBV mono-infected pregnancy, there have been several observational studies published in this area^2, 3, 10, 11, 21–24^. Recently, an excellent systematic review involving 26 studies was conducted by Brown *et al*.^25^, in which these authors comprehensively evaluated the efficacy and safety of LAM, LdT and TDF used in pregnancy; but only 3 observational studies on TDF (all with small sample sizes) were included in the study, and the results were limited^2, 3, 23^. Since then, a multi-center, randomized controlled trial was published to powerfully demonstrate the efficacy and safety profile of TDF in highly viremic CHB mothers^8^; however, according to the FDA drug category for pregnancy, TDF is still classified as category B for use in pregnancy, and therefore, guidelines form the WHO^14^, AASLD^17^ and APASL (2015 update)^16^ made this issue a priority for the development of clinical practice guidelines and the synthesis of evidence. To maximize sample size and add the latest evidence, we performed a systematic review and meta-analysis to comprehensively evaluate the efficacy of oral TDF therapy in preventing vertical transmission of hepatitis B in pregnancies carrying a high viral load.

## Results

**Figure 1:**
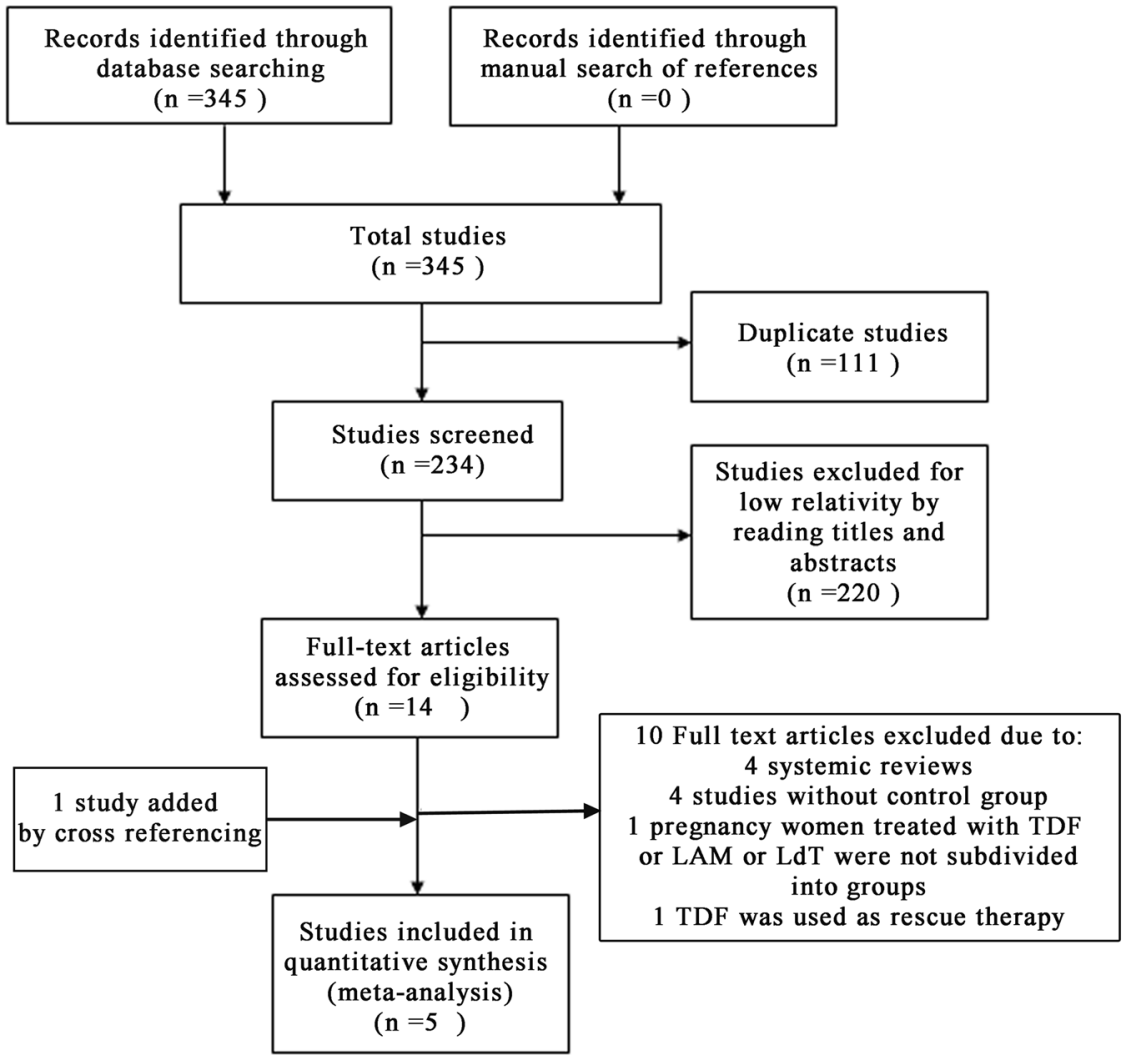
Flowchart of study selection^35^.

A flow diagram summarizing the process of study selection is illustrated in Fig. 1. A total of 345 potential studies published through July 31, 2016 were identified in a preliminary search of literature databases. After removing duplicates and initially screening publications by browsing their titles and abstracts, 16 publications (including 4 systemic reviews) regarding the efficacy and safety of TDF used in CHB mono-infected patients were identified; then we conducted further screening by browsing the full texts of the remaining publications, and reference lists to these articles were also manually searched, such that there were no studies conducted on the same populations. Finally, 5 studies were included according to our inclusion criteria, and the excluded articles and reasons for exclusion of each article are shown in Supplementary Table S1.

### Characteristics of included studies

Five studies, including 4 observational studies^2, 3, 22, 23^ and 1 RCT^8^, involving 585 pregnant women and 595 newborns, were included in the present meta-analysis. These very recent studies were published from 2013 to 2016; 2 studies were conducted in China^8, 23^, and another 3 studies were conducted in North American^22^, Australia^2^ and Turkey^3^, respectively. The participants in all studies in the TDF group received 300 mg of TDF once daily, with a baseline HBV DNA level of > 7.7 (range, 7.7–8.28) log10 IU/mL, and an average baseline ALT level of 30.5 U/L. In 4 studies TDF treatment was initiated from gestational weeks 30–32 until 4–12 weeks following delivery^2, 8, 22, 23^, while in the remaining studies TDF treatment was initiated from gestational weeks 18–27 until 4 weeks following delivery^3^. The pregnant women in the control group in all studies did not receive antiviral therapy. In 4 studies the investigators compared TDF *versus* control^3, 8, 22, 23^, while in 1 study researchers compared TDF *versus* LAM or control^2^. All infants in the included studies received a 3-dose HBV vaccination and hepatitis B immune globulin (HBIG) administration after delivery, and all mothers in these studies were instructed not to breast-feed their infants during the period in which they were receiving TDF treatment. More characteristics of the studies and baseline information on HBIG + HBV vaccine administered to infants are summarized in Tables 1 and 2, respectively. The overall quality of the included studies was adequate, and the bias risk for the included RCT study was low; while the mean score for 4 included observational studies was 8 (Table 3).

**Table 1:** Characteristics of the 5 included studies.

Author (Year)	Country	Compa-rison	Participants (mothers)	Infants	Age (years; mean, range or SD)	Dosage (TDF)	Recruit-ment period	Treatment Start (Gestational Weeks)	Treatment Disconti-nuation (Postpartum)	Follow-up of mothers (Postpartum)	Follow-up of infants	Baseline ALT Level (U/L; mean, range or SD)	Baseline of HBV-DNA level (log10 IU/ml; mean, range, or SD)	Study design
Samadi *et al*. (2016)	Canada	TDF *vs*	23	24	30 (28–34)	300 mg daily	01.2011 to	28–32	3 months	3 months	7–9 months	30.0 (18–50)	7.7 (3.2–8.1)	prospective
		control	138	146	32 (29–36)		12.2014					17.0 (12–24)	2.3 (1.6–3.1)	single-center non-RCT
Pan *et al*. (2016)	China	TDF *vs*	95	92	27.4 ± 3.0	300 mg daily	03.2012 to	30–32	4 weeks	28 weeks	28 weeks	15.0 (12.0–21.0)	8.19 (7.96–8.47)	prospective
		control	88	88	26.8 ± 3.0		6.2013					17.0 (11.0–22.2)	8.18 (7.72–8.51)	multi-center RCT
Chen *et al*. (2015)	China	TDF *vs*	62	66	32.41 ± 3.12	300 mg daily	2011–2013	30–32	1 month	6 months	12 months	23.27 ± 36.2	8.25 ± 0.45	prospective
		control	56	57	32.45 ± 3.20							16.59 ± 14.43	8.24 ± 0.35	multi-center non-RCT
Greenup *et al*. (2014)	Australia	TDF *vs*	58	58	30 ± 8.5	300 mg daily	2007–2010	32	12 weeks	48 weeks	9 months	28 (22–36)	7.94 ± 0.78	prospective
		LAM or	52	53	28 ± 5.3			32	12 weeks	48 weeks		25 (17–31)	7.72 ± 0.61	multi-center non-RCT
		control	20	20	28 ± 5								8 ± 0.04	
Celen *et al*. (2013)	Turkey	TDF *vs*	21	21	28.2 ± 4.1	300 mg daily	02.2010 to	18–27	4 weeks	28 weeks	28 weeks	56 (22–71)	8.28	Retrospective multi-center non-RCT
		control	24	23	26.9 ± 2.9		1.2012					52 (19–77)	8.31	

Abbreviations: TDF, tenofovir disoproxil fumarate; LAM, lamivudine; ALT, alanine aminotransferase; HBIG, hepatitis B immune globulin; NA, not available.

**Table 2:** Baseline information for HBIG + HBV vaccine given to infants in the 5 included studies.

Author (Year)	Groups	Dosage		Vaccination time (months)		Manufacturer	
		HBIG	HBV-vaccine	HBIG	HBV-vaccine	HBIG	HBV-vaccine
Samadi *et al*. (2016)	All infants	NA	NA	0	0,2,6	NA	NA
Pan *et al*. (2016)	All infants	200 IU	10 μg	0	0,1.6	GlaxoSmithKline	GlaxoSmithKline
Chen *et al*. (2015)	All infants	100 IU	20 μg	0	0,1,6	GlaxoSmithKline	GlaxoSmithKline
Greenup *et al*. (2014)	All infants	100 IU	10 μg	0	0,2,4,6	CSL Bioplasma	GlaxoSmithKline
Celen *et al*. (2013)	All infants	200 IU	20 μg	0	1,2,6	Talecris Biotherapeutic	Merck Sharp and Dohme

Abbreviations: HBIG, hepatitis B immune globulin; NA, not available.

**Table 3:** Risk of bias assessment for the included studies.

^a^ **RCTs**									
Author (Year)	Sequence generation	Allocation concealment		Blinding of participants, personnel, and assessors	Incomplete outcome data	Selective outcome reporting	Other sources of bias		Risk of bias
Pan *et al*. (2016)	Randomization table	Blocks and randomized		No blinding	No missing outcome data	All prespecified outcomes reported	No		Low
^b^ **Observational Studies**									
		Selection			Comparability		Outcome		Total score^c^
Author (Year)	Representativeness of the exposed cohort	Selection of the Non-exposed cohort	Ascertainment of exposure	Demonstration that outcome of interest was not present at start of study	Comparability of cohorts on the basis of the design or analysis	Assessment of outcome	Was follow-Up Long Enough for outcomes to occur?	Adequacy of cohorts Follow-Up	
Samadi *et al*. (2016)	Somewhat representative of the community or population	Drawn from the same community as the exposed cohort	Secure record	Yes	Study controls for any additional factors	Record linkage	Yes	Adequate	☆☆☆☆☆☆☆☆
Chen *et al*. (2015)	Somewhat representative of the community or population	Drawn from the same community as the exposed cohort ion	Secure record	Yes	Study controls for any additional factors	Record linkage	Yes	Adequate	☆☆☆☆☆☆☆☆
Greenup *et al*. (2014)	Somewhat representative of the community or population	Drawn from the same community as the exposed cohort	Secure record	Yes	Study controls for any additional factors	Record linkage	Yes	Adequate	☆☆☆☆☆☆☆☆
Celen *et al*. (2013)	Somewhat representative of the community or population	Drawn from the same community as the exposed cohort	Secure record	Yes	Study controls for any additional factors	Record linkage	Yes	Adequate	☆☆☆☆☆☆☆☆

^a^For RCTs, risk of bias was assessed with Cochrane Risk of Bias assessment tool.

^b^For observational studies, risk of bias was assessed with the Newcastle-Ottawa Scale.

^c^Calculated by adding the points awarded for each item.

### Maternal outcomes

**Figure 2:**
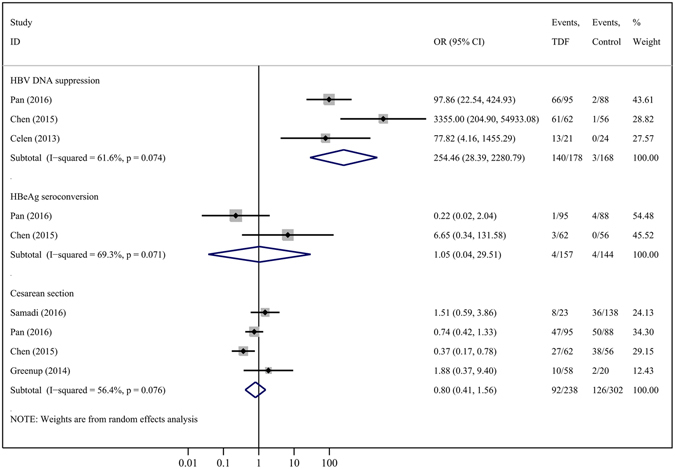
Forest plot showing meta-analysis of maternal outcomes for studies comparing TDF *versus* controls at delivery, based upon random-effects model.

**Figure 3:**
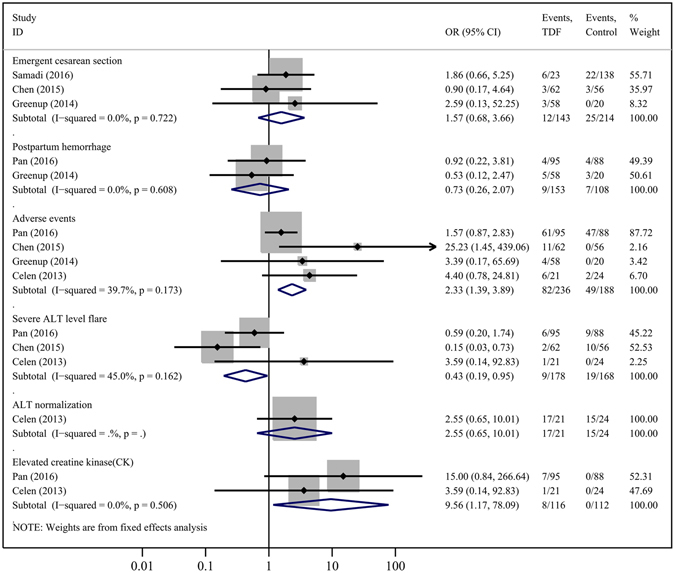
Forest plot showing meta-analysis of maternal outcomes for studies comparing TDF *versus* control at delivery, based upon fixed-effects model.

Compared to controls, TDF treatment significantly improved HBV DNA suppression at delivery (3 studies, OR = 254.46, 95% CI = 28.39–2280.79; P = 0.000) (Fig. 2). Two studies depicted the efficacy of TDF on maternal HBeAg seroconversion; however, no significant difference was found between the 2 groups in this index (OR = 1.05, 95% CI = 0.04–29.51; P = 0.979) (Fig. 2), and the HBV DNA levels in the treatment group gradually increased and became comparable to those of the control group at 1 to 6 months after the discontinuation of TDF^8, 23^. However, in the study conducted by Celen *et al*.^3^, 61.9% (13/21) of mothers still showed HBV DNA < 50 IU/mL at 28 weeks postpartum. Regarding the influence of maternal serum ALT levels, synthesis results from 3 studies suggested that TDF generated a less-severe flare in ALT levels (OR = 0.43, 95% CI = 0.19–0.95; P = 0.038) during pregnancy (Fig. 3). However, no significant difference was found in maternal ALT normalization at delivery (1 cohort, OR = 2.55, 95% CI = 0.65–10.01; P = 0.180) (Fig. 3).

When comparing TDF therapy with respect to controlling for maternal harm, there were no statistical differences found in the rates of cesarean section (4 studies, OR = 0.80, 95% CI = 0.41–1.56; P = 0.521) (Fig. 2), emergent cesarean section (3 studies, OR = 1.57, 95% CI = 0.68–3.66; P = 0.291) (Fig. 3), or postpartum hemorrhage (2 studies, OR = 0.73, 95% CI = 0.26–2.07; P = 0.549) (Fig. 3). Although TDF induced more drug-related adverse events (4 studies, OR = 2.33, 95% CI = 1.39–3.89; P = 0.001) and elevated CK (2 studies, OR = 9.56, 95% CI = 1.17–78.09; P = 0.035) compared with controls (Fig. 3), almost all of the TDF-related adverse events and elevations in CK levels were grades 1 or 2, including fatigue, headache, cough, diarrhea, fever, nausea, pruritus, palpitation, dyspepsia, rash, insomnia, dizziness, abdominal pain, jaundice, and upper respiratory infection; however, all of these adverse events were mild or moderate, and symptomatic therapy or a temporary withdrawal of TDF alleviated adverse events and/or the patients could return to normal activities.

**Figure 4:**
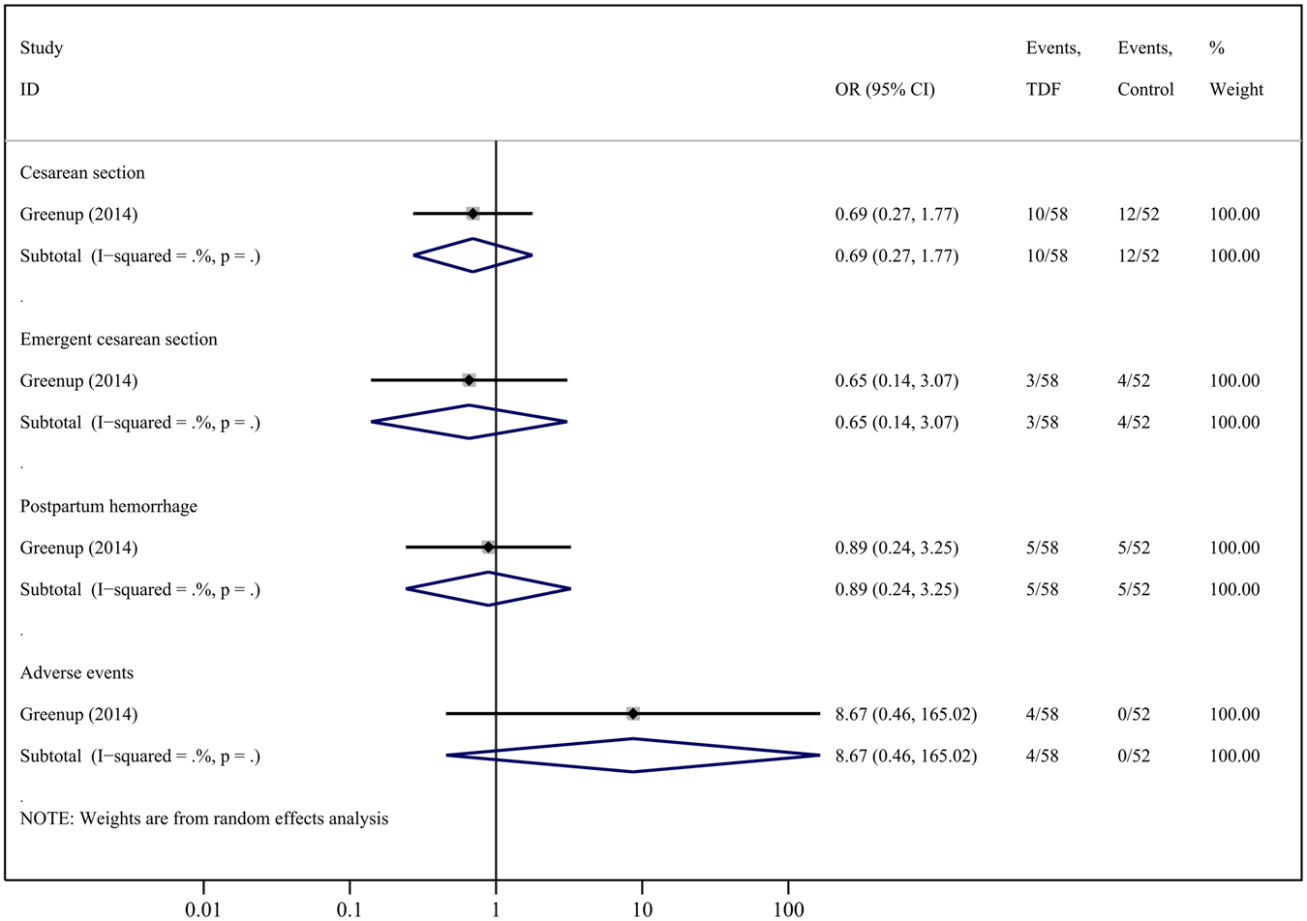
Forest plot showing meta-analysis of maternal outcomes for studies comparing TDF *versus* LAM at delivery, based upon random-effects model.

In the study comparing TDF to LAM^2^, there was no difference found in any of several maternal outcomes (Fig. 4); however, the data here were limited.

The quality of the evidence regarding maternal outcomes was low to very low due to the observational nature of the studies, imprecision, and indirectness; and moderate for adverse events due to imprecision. The online Supplementary Table S3 summarizes the quality of evidence (GRADE) for infant and maternal outcomes.

### Infant outcomes

**Figure 5:**
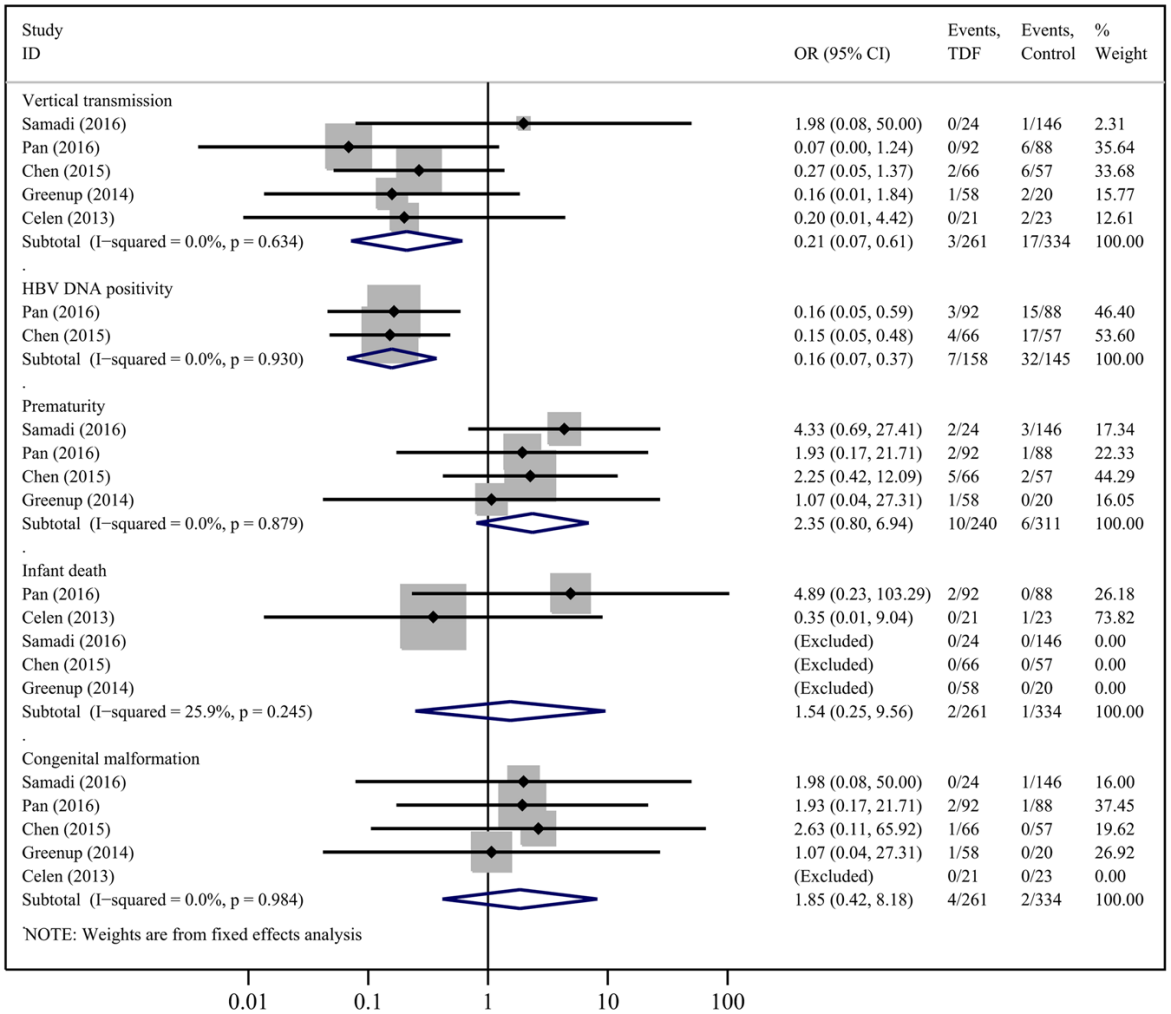
Forest plot showing meta-analysis of infant outcomes for studies comparing TDF *versus* control, based upon fixed-effects model.

As shown in Fig. 5, our analysis showed that infants born from pregnant women treated with TDF had a significantly lower vertical transmission rate compared to those born from the control group, as defined by infant HBsAg seropositivity at 4–12 months (5 studies, OR = 0.21, 95% CI = 0.07–0.61; P = 0.004), and TDF showed better improvement in reducing infant HBV DNA seropositivity at delivery (2 studies, OR = 0.16, 95% CI = 0.07–0.37; P = 0.000). TDF also significantly reduced the risk of infant HBV DNA seropositivity at delivery by 17.6%, and 1.36%-18% of newborns failed HBV immunoprophylaxis^2, 3, 8, 22, 23^ in the control group.

**Figure 6:**
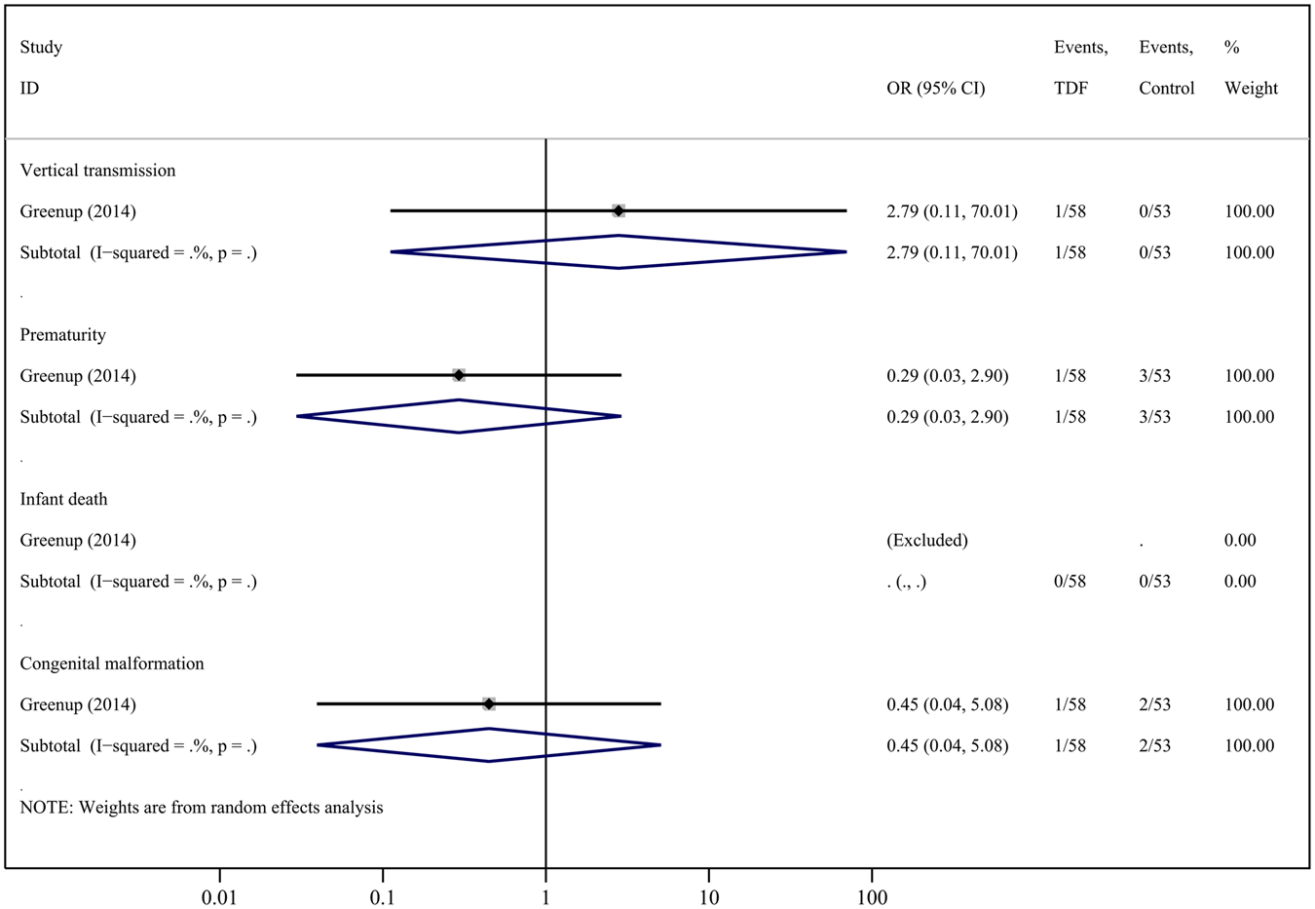
Forest plot showing meta-analysis of infant outcomes for studies comparing TDF *versus* LAM, based upon random-effects model.

Compared to controls, TDF therapy did not show any statistically significant differences in rates of prematurity (4 cohorts, OR = 2.35, 95% CI = 0.80–6.94; P = 0.121), infant death (5 cohorts, OR = 1.54, 95% CI = 0.25–9.56; P = 0.644), or congenital malformations (5 cohorts, OR = 1.85, 95% CI = 0.42–8.18; P = 0.420). The congenital malformations observed in these studies were congenital chromosomal disorder syndrome^22^, polydactyly ^2,23^, unilateral deafness and absent ear^2^, torticollis, umbilical hernia and hypospadias^8^. In the study comparing TDF and LAM^2^, the authors observed no differences in any of several infant outcomes (Fig. 6); however, these data were again limited.

The quality of the evidence regarding infant outcomes was low to very low due to the observational nature of the studies, imprecision, and indirectness (see Supplementary Table S2 online).

### Publication bias

We were unable to evaluate publication bias due to the small number of studies available for each outcome.

## Discussion

CHB infection remains a major challenge to global health, and by blocking the routes of HBV transmission, global health rates should improve. Vertical transmission is currently the predominant mode of CHB infection, and a linear association between vertical transmission and maternal viral load levels has been demonstrated in several studies^9, 13, 17^. Additionally, 8%-30% of newborns who were born to pregnant women with a high serum HBV-DNA level of > 2 × 10^5^–10^7^ IU/mL^8–13^ showed failed immunoprophylaxis. However, vertical transmission also did not occur in pregnant women with HBV-DNA levels < 2 × 10^5^ IU/mL, as reported in a few studies^13, 26^. Thus, AASLD guidelines now recommend an HBV DNA level of > 2 × 10^5^ IU/mL, but not ≤ 2 × 10^5^ IU/mL, as the threshold to initiate antiviral therapy in the prevention of vertical transmission, based on low-quality existing evidence^17^. Women who are infected with CHB in their childbearing years may require antiviral therapy, and those women being treated for CHB may become pregnant. Unfortunately, the efficacy and safety of antivirals during pregnancy (especially their impact on potential teratogenicity) has not yet been proven; and there have been no identifiable antiviral drugs recommended to reduce vertical transmission of HBV. Thus, characterizing the efficacy and safety of these medications for mother and infant during pregnancy would help inform optimization and potential treatment choices for women of childbearing age.

Brown and colleagues^25^ recently carried out an excellent systematic review involving 26 studies that comprehensively evaluated the efficacy and safety of LAM, LdT and TDF used in pregnancy; and their results revealed that use of LAM and LdT significantly reduced vertical transmission and they were safe in pregnant women, with no increase in adverse maternal or fetal outcomes. These same authors identified no safety issues with TDF use in maternal or fetal outcomes, possibly because their meta-analysis contained an insufficient amount of controlled outcome data. In the present systematic review, we added the latest evidence to clearly delineate the benefits and risks associated with TDF use in pregnant women infected with CHB at a high viral load: synthesis results revealed that TDF was effective and safe in improving maternal HBV DNA suppression at delivery and in reducing fetal HBsAg seropositivity at 4–12 months or HBV DNA seropositivity at delivery. Most importantly, TDF significantly reduced vertical transmission at 4–12 months postpartum, without increasing rates of cesarean section, emergent cesarean section, postpartum hemorrhage of the mother; and prematurity, infant death, and congenital malformations in infants. In the current study, we also found that 1.36%-18% of newborns failed HBV immunoprophylaxis when their mothers were untreated, which was consistent with previously existing evidence^8–13^. In addition, maternal virus rebounded and became comparable to levels of the control group at 1 to 6 months after the discontinuation of TDF. The favorable safety profile of TDF has been well demonstrated in HIV mono-infected and HIV/HBV co-infected mothers^10, 18–20^; and all of these results, then, support the use of antiviral therapy to reduce maternal viral load and rescue infants from failed immunoprophylaxis.

There were insufficient controlled outcome data to compare TDF with other NAs in CHB-infected pregnant women with high viral load, and results from one included study suggested that there was no statistically significant difference between TDF and LAM on maternal and infant outcomes; however, due to the limitations of potency and low threshold to resistance of LAM and LdT, TDF remains a promising NA used in preventing vertical transmission. TDF is also approved as possessing the highest potency and is the only approved NA without any associated clinical resistance after up to 6 years of monotherapy for CHB^16, 27^. In fact, in recent studies, TDF has been demonstrated to be effective and safe in pregnant women with CHB who have LAM or LdT resistance^24, 28^. According to the FDA drug category for pregnancy, TDF and LdT are classified as category B for use in pregnancy; while LAM, ADV and ETV are classified as category C drugs based primarily on animal data^16^. Although breast-feeding by women who are on antiviral therapy is still controversial and not recommended, data from small studies in HIV-infected women have demonstrated that the median amount of TDF ingested from breast milk was only 0.03% of the recommended pediatric dose^29, 30^, and thus oral TDF appears safe and is recommended before using other NAs.

The conclusions of this systematic review should be treated with caution due to several limitations. First, studies warranting high confidence are absent from the present meta-analysis, with only one RCT; and most of the data are derived from observational studies, which are subject to significant biases–especially selection bias. Second, the quality of the evidence for all outcomes was low to very low due to the observational nature of the studies, imprecision, and indirectness. Third, several questions addressed in the previous AASLD HBV Guidelines were not resolved in this systematic review: a) What is the exact viral load threshold and the exact week within the third trimester at which therapy should be initiated?and b) Is it safe for infants to be exposed to antivirals late in pregnancy from longitudinal follow-up and breast-feeding while the mothers are on antiviral therapy? It has been speculated that the risk of HBV vertical transmission (despite active and passive immunoprophylaxis at birth), could be partially attributable to an unsatisfactory approach in terms of the timing of prophylaxis and quality of the vaccine adopted, and thus, c) Is there a relationship between the timing or quality of immunoprophylaxis and the risk of vertical transmission? None of these subjects has been well documented presently because data from controlled studies for these patient populations were sparse and require further corroboration in the future, especially in the form of high-quality, larger-scale RCTs.

In conclusion, oral TDF lowers HBV DNA levels in CHB-infected pregnant women as it does in non-pregnant women, and it reduces the rates of vertical transmission. These effects were demonstrated in CHB-infected pregnant women with high viral loads (>2 × 10^5^ IU/mL). The limited safety data suggest that there is no increased risk for adverse maternal or fetal outcomes; and therefore, in order to prevent vertical transmission, we conservatively recommend the use of TDF in pregnant women with CHB and who manifest high HBV DNA levels of > 2 × 10^5^ IU/mL in the third trimester.

## Materials and methods

### Literature search strategy

This systematic review was carried out according to the methodology developed by the Cochrane Collaborative Review Group, and we applied the PRISMA guidelines for the reporting of systematic reviews and meta-analyses^31^.

Studies published through July 2016 in which investigators evaluated the efficacy and safety of TDF in preventing vertical transmission of HBV in pregnancy were identified through systematic searches of PubMed, the OvidSP search platform, ACP Journal Club, Cochrane Central Register of Controlled Trials, Cochrane Database of Systematic Reviews, Cochrane Methodology Register, Database of Abstracts of Reviews of Effects, Embase, Epub Ahead of Print, In-Process & Other Non-Indexed Citations, Ovid MEDLINE(R) Daily and Ovid MEDLINE(R) 1946 to Present, Scopus, Chinese National Knowledge Infrastructure (CNKI), Chinese Biomedical literature database (CBM), and WANFANG databases, with no language limitations. The search strategy was designed and modified according to existing reviews^25^, and we queried experienced librarians from our affiliated school when necessary; then an optimized search strategy was conducted by the principal investigator. Reference lists in relevant original and review articles were also manually searched. Full details of the databases searched and that were used to identify the studies included in this meta-analysis are shown in Supplementary Table S3.

### Inclusion criteria

We included randomized controlled trials (RCTs) and controlled observational studies in this meta-analysis if they (a) enrolled pregnant women monoinfected with chronic HBV (characterized by the presence of HBsAg for more than 6 months), with high serum HBV-DNA levels > 6 log copies/ml before antiviral therapy; (b) compared the efficacy and safety profile of TDF with placebo or other NAs; (c) reported the outcomes of interest, including prevention of vertical transmission of HBV, clinical efficacy, and adverse outcomes from antiviral therapy to both mothers and newborns; and (d) provided sufficient information for estimating odds ratios (OR) and 95% confidence intervals (CI). However, studies that enrolled infants who did not receive immunization during the first week postpartum; or that enrolled patients who received steroids, chemotherapy/immunotherapy, liver transplantation, or hemodialysis; or unpublished reports and abstracts or published as abstracts only; or uncontrolled studies, were excluded^25^. In the case of recent studies, we included those entailing the largest number of patients, especially when studies had overlapping (or the same) sample sizes. Incomplete data or missing data and additional studies were obtained by contacting authors by email, telephone, or other mode of communication

### Data extraction

Two reviewers (JZC and ZWL) independently and in duplicate screened the titles and abstracts from the literature and identified eligible papers based upon the inclusion criteria. Next, the full texts of potentially eligible papers were further reviewed following the same procedure. Three reviewers (LFH, RKS and WBW) independently extracted the following data using a standardized, pretested Excel form: study characteristics, participant baseline characteristics, intervention details, and outcomes of interest. Discrepancies were resolved through discussion with a third author (ZH).

### Outcomes

Outcomes of interest were as follows: maternal outcomes including HBV DNA suppression, HBeAg seroconversion, alanine aminotransferase (ALT) normalization, severe flares of ALT levels (defined as an elevated ALT level > 5 times the upper limit of the normal range)^8^; rates of cesarean section, emergent cesarean section, postpartum hemorrhage, and adverse events; and elevated levels of creatine kinase (CK). Infant outcomes included vertical transmission (defined as HBsAg seropositivity at 6–12 months or HBV DNA positivity at 6–12 months), prematurity, congenital malformations, and infant death.

### Bias risk assessments

Two reviewers (JZC and ZWL) independently assessed the bias risk of RCTs with the Cochrane Risk of Bias assessment tool, and the Newcastle-Ottawa Scale for observational studies. The quality of evidence was evaluated using the Grading of Recommendations Assessment, Development, and Evaluation approach (GRADE); and criteria used to evaluate the quality of evidence were risk of bias, including indirectness (surrogate outcomes), imprecision (wide confidence intervals), inconsistency (heterogeneity), and publication bias^25, 32^.

### Statistical analysis

We calculated the odds ratios (ORs) and 95% confidence intervals (CIs) using the binomial distribution for dichotomized outcomes, and we planned to use the mean difference (MD) or standardized mean difference (SMD) between the baseline and the longest duration of follow-up for each study of continuous outcomes. Heterogeneity across studies was assessed by calculating I^2^: a value < 25% was considered low; 25–50%, moderate; and > 50%, high. We planned to calculate the pooled effect size using the (I-V heterogeneity) random-effects model when I^2^ > 50%, and a Mantel-Haenszel (M-H) fixed-effects model otherwise^33, 34^. The significance of pooled effect size was determined using the Z-test, with a 2-tailed P < 0.05 defined as the significance threshold.

If appropriate, we planned to repeat the meta-analysis after excluding individual studies to assess the stability of the results. If the included studies were unsuitable or insufficient, studies were identified for meta-analysis in terms of certain outcomes of interest, and we summarized the results qualitatively; if the included RCTs were insufficient, we planned to synthesize the results of RCTs with controlled observational studies. Publication bias was assessed using the Egger regression asymmetry test and Begg’s funnel plots when a sufficient number of studies (>20) per outcome were available and if heterogeneity was low^25^. All statistical tests for this meta-analysis were performed using STATA, version 12 (StataCorp LP, College Station, TX).

### Data Availability

All data generated or analysed during this study are included in this published article (and its Supplementary Information files).

## Electronic supplementary material

Supplementary Information
