# Supplementary material for: Efficacy and safety of tenofovir disoproxil fumarate in preventing vertical transmission of hepatitis B in pregnancies with high viral load
Source: Sci Rep. 2017 Jun 23;7:4132. doi: 10.1038/s41598-017-04479-x (PMC5482834; doi:10.1038/s41598-017-04479-x)
Supplement: Supplementary file 1 — Supplementary Information [file 41598_2017_4479_MOESM1_ESM.doc]

**Efficacy and safety of tenofovir disoproxil fumarate in preventing vertical transmission of hepatitis B in pregnancies with high viral load**

Jun-Ze Chen^1#*^ Zuo-Wei Liao^1#^ Fei-Long Huang^1^ Ru-Kui Su^1^ Wen-Bo Wang^1^ Xue-Yuan Cheng^1^ Jie-Qing Chen^1^ Jia-Qi Liu^1^ Zhong Huang^1*^

1. Department of General Surgery, The Ninth Affiliated Hospital of Guangxi Medical University, Beihai, 536000, PR China

# Co-first authors.

* Correspondence and requests for materials should be addressed to:

Jun-Ze Chen (Tel: +86-186-0779-0368; E-mail: junzechen89@163.com) and Zhong Huang; (Tel: +86-139-7795-8890; E-mail: hzhong8890@126.com)

**Supplementary Table S1.** The excluded articles and reasons for exclusion of each article.

| Author | Title of the excluded articles | Reasons for exclusion |
| --- | --- | --- |
| Wang *et al*.[^1^](#_ENREF_1) | Cost-effectiveness of antiviral therapy during late pregnancy to prevent perinatal transmission of hepatitis B virus | systematic review and meta-analysis |
| Njei *et al*.[^2^](#_ENREF_2) | TDF Comparative efficacy of antiviral therapy in preventing vertical transmission of hepatitis B: A network meta-analysis | systematic review and meta-analysis |
| Brown *et al*.[^3^](#_ENREF_3) | Antiviral therapy in chronic hepatitis B viral infection during pregnancy: A systematic review and meta-analysis | systematic review and meta-analysis |
| Khungar *et al*.[^4^](#_ENREF_4) | A systematic review of side effects of nucleoside and nucleotide drugs used for treatment of chronic hepatitis B | systematic review and meta-analysis |
| Wang *et al*.[^5^](#_ENREF_5) | Efficacy of tenofovir disoproxil fumarate to prevent vertical transmission in mothers with lamivudine-resistant HBV | All participants treated with TDF, there was no control group in study design. |
| Chen *et al*.[^6^](#_ENREF_6) | Efficacy of maternal tenofovir disoproxil fumarate in interrupting mother-to-infant transmission of hepatitis B virus | All participants treated with TDF, there was no control group in study design. |
| Pan *et al*.[^7^](#_ENREF_7) | Tenofovir disoproxil fumarate for prevention of vertical transmission of hepatitis B virus infection by highly viremic pregnant women: a case series | All participants treated with TDF, there was no control group in study design. |
| Fu *et al*.[^8^](#_ENREF_8) | Safety and efficacy of tenofovir disoproxil in treatment of women with chronic hepatitis B during  pregnancy | All participants treated with TDF, there was no control group in study design. |
| Tekin Koruk *et al*.[^9^](#_ENREF_9) | Evaluation of hepatitis B virus transmission and antiviral therapy among hepatitis B surface antigen-positive pregnant women | Pregnant women treated with TDF or LAM or LdT were not subdivided into groups. |
| Hu *et al*.[^10^](#_ENREF_10) | Tenofovir rescue therapy in pregnant females with chronic hepatitis B | TDF was used as rescue therapy |

**References**

**Supplementary Table S2.** Quality of evidence summary.

| Intervention | Outcome  (Follow up) | No. of participants  (Study design) | Quality of the evidence  (GRADE) | Relative effect  (95% CI) |
| --- | --- | --- | --- | --- |
| Maternal outcomes | | | | |
| TDF  vs  Control | HBN DNA suppression  (at delivery) | 346  (3 studies: 1RCT and 2 observational studies) | ⊕⊕⊝⊝ LOW^3,4^ | OR 254.461 (28.390-2280.788) |
|  | HbeAg seroconversion  (at delivery) | 301  (2 studies: 1RCT and 1 observational studies) | ⊕⊝⊝⊝ VERY LOW^2,3,4^ | OR 1.047  (0.037-29.507) |
|  | Cesarean section | 540  (4 studies: 1RCT and 3 observational studies) | ⊕⊕⊝⊝ LOW^3,4^ | OR 0.805  (0.415-1.563) |
|  | Emergent cesarean section | 357  (3 observational studies) | ⊕⊕⊝⊝ LOW^3,4^ | OR 1.575  (0.678-3.658) |
|  | Postpartum hemorrhage | 261  (2 observational studies) | ⊕⊝⊝⊝ VERY LOW^2,3,4^ | OR 0.726  (0.255-2.069) |
|  | Adverse events | 424  (4 studies: 1RCT and 3 observational studies) | ⊕⊕⊕⊝  MODERATE^4^ | OR 2.327  (1.393-3.890) |
|  | Severe ALT Level flare  (at delivery) | 346  (3 studies: 1RCT and 2 observational studies) | ⊕⊕⊝⊝ LOW^3,4^ | OR 0.429  (0.193-0.952) |
|  | ALT normalization  (at delivery) | 45  (1 observational study) | ⊕⊝⊝⊝ VERY LOW^2,3,4^ | OR 2.550  (0.650-10.007) |
|  | Elevated creatine kinase(CK) | 346  (2 studies: 1RCT and 1 observational study) | ⊕⊕⊝⊝ LOW^3,4^ | OR 9.556  (1.169-78.094) |
| TDF  vs  LAM | Cesarean section | 110  (1 observational studiesy) | ⊕⊝⊝⊝ VERY LOW^2,3,4^ | OR 0.694  (0.272-1.775) |
|  | Emergent cesarean section | 110  (1 observational study) | ⊕⊝⊝⊝ VERY LOW^2,3,4^ | OR 0.655  (0.139-3.072) |
|  | Postpartum hemorrhage | 110  (1 observational study) | ⊕⊝⊝⊝ VERY LOW^2,3,4^ | OR 0.887  (0.242-3.255) |
|  | Adverse events | 110  (1 observational study) | ⊕⊝⊝⊝ VERY LOW^2,3,4^ | OR 8.670  (0.455-165.019) |
| **Infant outcomes** | | | | |
| TDF  vs  Control | Vertical transmission  (at 4-12 months) | 595  (5 studies: 1RCT and 4 observational studies) | ⊕⊕⊝⊝ LOW^3,4^ | OR 0.210  (0.072-0.611) |
|  | HBV DNA positivity  (at delivery) | 303  (2 studies: 1RCT and 1 observational studies) | ⊕⊕⊝⊝ LOW^3,4^ | OR 0.157  (0.067-0.372) |
|  | Prematurity rate | 551  (4 studies: 1RCT and 3 observational studies) | ⊕⊕⊝⊝ LOW^3,4^ | OR 2.353  (0.798-6.936) |
|  | Infant death rate | 595  (5 studies: 1RCT and 4 observational studies) | ⊕⊕⊝⊝ LOW^3,4^ | OR 1.538  (0.247-9.556) |
|  | Congenital malformation | 595  (5 studies: 1RCT and 4 observational studies) | ⊕⊕⊝⊝ LOW^3,4^ | OR 1.846  (0.416-8.180) |
| TDF  vs  LAM | Vertical transmission  (at 4-12 months) | 111  (1 observational study) | ⊕⊝⊝⊝ VERY LOW^2,3,4^ | OR 2.791  (0.111-70.014) |
|  | Prematurity rate | 111  (1 observational study) | ⊕⊝⊝⊝ VERY LOW^2,3,4^ | OR 0.292  (0.029-2.901) |
|  | Infant death rate | 111  (1 observational study) | ⊕⊝⊝⊝ VERY LOW^2,3,4^ | NA |
|  | Congenital malformation | 111  (1 observational study) | ⊕⊝⊝⊝ VERY LOW^2,3,4^ | OR 0.447  (0.039-5.082) |

1. Increased risk of bias; 2. Inconsistency; 3. Indirectness; 4. Imprecision

Abbreviations: TDF: tenofovir disoproxil fumarate ; LAM: lamivudine ; ALT: alanine aminotransferase; GRADE: Grading of Recommendations Assessment, Development, and Evaluation approach; NA: Not Available.

**Supplementary Table S3.** Full details of the search strategies to identify the studies included in this meta-analysis.

| Databases | Search strategies | Records in each step |
| --- | --- | --- |
| Pubmed  (84 records) | Search in all fields:  #10 ((#9) AND #6) AND #3  #9 (#7) OR #8  #8 ((tenofovir or “tenofovir disoproxil” or “tenofovir disoproxil fumarate” or TDF))  #7 tenofovir[MeSH Terms]  #6 (#4) OR #5  #5 ("hepatitis B" or "serum hepatitis" or "hippie hepatitis" or "injection hepatitis" or "hepatitis type B" or "hepatitis B virus" or "chronic hepatitis B" or HBV or CHB)  #4 hepatitis B[MeSH Terms]  #3 (#1) OR #2  #2  (pregnan* or gestation* or "child bearing" or childbearing or intrauterine or maternity or mother or perinatal or maternal)  #1 pregnancy[MeSH Terms] | 84  5,728  5,728  2,696  93,140  93,140  49,897  1,138,040  1,128,072  789,651 |
| Ovid  Database(s):  EBM Reviews - ACP Journal Club 1991 to June 2016;  EBM Reviews - Cochrane Central Register of Controlled Trials June 2016;  EBM Reviews - Cochrane Database of Systematic Reviews 2005 to July 21, 2016;  EBM Reviews - Cochrane Methodology Register 3rd Quarter 2012;    EBM Reviews - Database of Abstracts of Reviews of Effects 1st Quarter 2016;  Embase 1974 to 2016 July 21;  Epub Ahead of Print, In-Process & Other Non-Indexed Citations, Ovid MEDLINE(R) Daily and Ovid MEDLINE(R) 1946 to Present  (76 records) | 1 exp Pregnancy/  2 (pregnan* or gestation* or "child bearing" or childbearing or intrauterine or maternity or mother or perinatal or maternal).mp.  3 1 or 2  4 exp hepatitis B/  5 ("hepatitis B" or "serum hepatitis" or "hippie hepatitis" or "injection hepatitis" or "hepatitis type B" or "hepatitis B virus" or "chronic hepatitis B" or HBV or CHB).mp.  6 4 or 5  7 3 and 6  8 exp tenofovir/  9 exp tenofovir disoproxil/  10 (tenofovir or tenofovir disoproxil or tenofovir disoproxil fumarate or TDF).mp.  11 8 or 9 or 10  12 7 and 11  13 randomized controlled trial.pt.  14 controlled clinical trial.pt.  15 randomized.ab.  16 placebo.ab.  17 drug therapy.fs.  18 randomly.ab.  19 trial.ab.  20 groups.ab.  21 13 or 14 or 15 or 16 or 17 or 18 or 19 or 20  22 exp animals/ not humans.sh.  23 21 not 22  24 12 and 23 | 1,475,813  2,351,433  2,361,964  133,150  233,848  233,848  12,761  15,996  6,333  27,068  27,068  541  820,893  179,249  1,128,169  560,932  5,204,508  717,902  1,089,907  3,987,047  10,184,316  26,195,666  4,495,113  76 |
| Scopus  (182 records) | #5 #1 and #2 and #3 and #4  #4 TITLE-ABS-KEY ( "randomized controlled trial"  OR  "controlled clinical trial"  OR  randomized OR  placebo  OR  "drug therapy"  OR  randomly  OR  trial  OR  groups )  #3 TITLE-ABS-KEY((tenofovir or "tenofovir disoproxil" or "tenofovir disoproxil fumarate" or TDF))  #2 TITLE-ABS-KEY( ("hepatitis B" or "serum hepatitis" or "hippie hepatitis" or "injection hepatitis" or "hepatitis type B" or "hepatitis B virus" or "chronic hepatitis B" or HBV or CHB))  #1 TITLE-ABS-KEY( (pregnan* or gestation* or "child bearing" or childbearing or intrauterine or maternity or mother or perinatal or maternal)) | 182  8,047,920  17,085  123,278  1,388,496 |
| Chinese National Knowledge Infrastructure(CNKI), and the WANFANG and VIP databases.  (3 records) | Chinese words:  (pregnancy OR pregnancy woman) and (tenofovir disoproxil fumarate) and (hepatitis B OR chronic hepatitis B) | 3 |
